# Cerebral Reorganization in Subacute Stroke Survivors after Virtual Reality-Based Training: A Preliminary Study

**DOI:** 10.1155/2017/6261479

**Published:** 2017-06-28

**Authors:** Xiang Xiao, Qiang Lin, Wai-Leung Lo, Yu-Rong Mao, Xin-chong Shi, Ryan S. Cates, Shu-Feng Zhou, Dong-Feng Huang, Le Li

**Affiliations:** ^1^Department of Rehabilitation Medicine, Guangdong Engineering Technology Research Center for Rehabilitation Medicine and Clinical Translation, The First Affiliated Hospital, Sun Yat-sen University, Guangzhou, China; ^2^Department of Rehabilitation Medicine, Luohu People's Hospital, Shenzhen, China; ^3^Department of Nuclear Medicine, The First Affiliated Hospital, Sun Yat-sen University, Guangzhou, China; ^4^Department of Pharmaceutical Sciences, College of Pharmacy, University of South Florida, Tampa, FL, USA

## Abstract

**Background:**

Functional magnetic resonance imaging (fMRI) is a promising method for quantifying brain recovery and investigating the intervention-induced changes in corticomotor excitability after stroke. This study aimed to evaluate cortical reorganization subsequent to virtual reality-enhanced treadmill (VRET) training in subacute stroke survivors.

**Methods:**

Eight participants with ischemic stroke underwent VRET for 5 sections per week and for 3 weeks. fMRI was conducted to quantify the activity of selected brain regions when the subject performed ankle dorsiflexion. Gait speed and clinical scales were also measured before and after intervention.

**Results:**

Increased activation in the primary sensorimotor cortex of the lesioned hemisphere and supplementary motor areas of both sides for the paretic foot (*p* < 0.01) was observed postintervention. Statistically significant improvements were observed in gait velocity (*p* < 0.05). The change in voxel counts in the primary sensorimotor cortex of the lesioned hemisphere is significantly correlated with improvement of 10 m walk time after VRET (*r* = −0.719).

**Conclusions:**

We observed improved walking and increased activation in cortical regions of stroke survivors after VRET training. Moreover, the cortical recruitment was associated with better walking function. Our study suggests that cortical networks could be a site of plasticity, and their recruitment may be one mechanism of training-induced recovery of gait function in stroke. This trial is registered with ChiCTR-IOC-15006064.

## 1. Introduction

Gait impairment is a common consequence of stroke, and the decreases in gait velocity, stride length, and cadence are hallmark features of gait pattern alterations in stroke survivors [1, 2]. Previous studies found that early intervention with physical therapy and gait training to restore walking after stroke was recommended to improve motor function and decrease disability [3, 4]. As gait impairments are a result of deficient neuromuscular control, a better understanding of the impact and mechanism of those interventions on gait pattern recovery after stroke is therefore essential.

Environmental factors act as critical determinants for the level of community ambulation of stroke patient [5]. The development of computers has resulted in virtual reality (VR) tools which can create life-like scenarios via visual, auditory, and tactile feedback and can provide subjects with a safe and stimulating learning environment [6]. VR has been increasingly used in poststroke rehabilitation; therapy interventions using VR may improve motor function for those patients [7–15]. VR system might represent the main neural substrate for relearning or resuming impaired motor functions following stroke. A key challenge in neurorehabilitation is to establish optimal training protocols for the given patient [10]. VR could provide a person with senses of encouragement and accomplishment [16–19]. However, two main concerns need to be investigated. What kind of rehabilitation strategies can combine with VR, and what degree for those VR combined rehabilitation strategies can facilitate stroke patients? Recently, motor relearning strategies can be applied in VR-enhanced treadmill (VRET) training by numerous movement repetitions and a multisensory approach to stimulate brain plasticity and patients receive visual feedback which is close to real-life experience [12]. While the positive benefits of VRET exercise on gait speed, cadence, step length, community walking time, and balance have been demonstrated [7–9, 11, 12, 14, 15], the associated changes of brain activity with this training have not been investigated yet.

Advances in imaging, such as blood oxygenation level-dependent functional magnetic resonance imaging (fMRI), have been allowed for the observation of changes in cerebral plasticity and the exploration of recovery mechanisms. The control of gait involves the planning and execution from multiple cortical areas, such as secondary and premotor cortex [11]. Ankle dorsiflexion is an important kinematic aspect of the gait cycle. Using ankle movement, Enzinger et al. [20] observed increased activation in the unlesioned hemisphere associated with increasing functional impairment of the paretic leg in patients with stroke. fMRI studies of patients after stroke have suggested that VR could increase neural activations in the primary motor areas and improve lateralization of primary sensorimotor cortex (SMC) activity [21–23]. We hypothesized that recovery of lower limb function after VRET would be associated with changes in brain activation during ankle dorsiflexion.

Therefore, the primary aim of this preliminary study was to investigate if functional reorganization takes place after VRET in subacute stroke survivors with gait impairment, using fMRI and an ankle dorsiflexion paradigm. Correlation between clinical scale changes after VERT and brain activation alterations was also studied to see the relations of the induction of cortical plasticity and functional recovery in subacute stroke survivors. We hope that the results of the current study could help to understand the mechanism of VRET as an early intervention for gait recovery for stroke.

## 2. Methods

### 2.1. Participants

**Figure 1:**
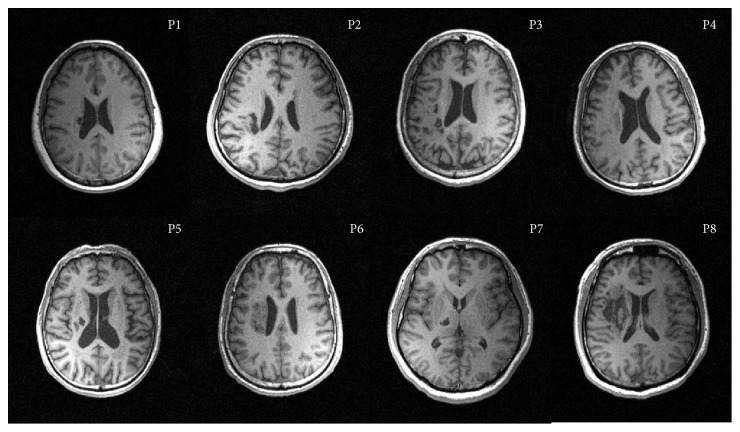
Axial structural T1-weighted MRI scans at the level of maximum infarct volume for each patient. And right hemisphere patients flipped on the sagittal axis for better comparison.

Eight stroke survivors were recruited in this study, aged 41–72 years (mean: 58.38 years) and included 6 males and 2 females (Table 1 and Figure 1). Inclusion criteria: (i) 18 to 80 years in ages; (ii) right-foot dominant; (iii) first incident of ischemic cortical or subcortical stroke which resulted in gait impairment; (iv) stroke was confirmed by MRI within the past 3 months of inclusion; (v) at least 10° of dorsiflexion is available at the ankle. Exclusion criteria: (i) contraindication to MRI scan (implanted medical devices incompatible with MRI testing or claustrophobia); (ii) history of stroke resulted in function impairment; (iii) history of mental disorder or the use of antipsychotic medication; (iv) cognitive impairment (Mini-Mental State Examination score of less than 24 points); (v) unable to speak or hear; (vi) history of recent deep vein thrombosis of the lower limbs; (vii) recent myocardial infarction; (viii) medically unstable; (ix) existing lower extremity pathology. This study was approved by the Ethics Committee of the First Affiliated Hospital of Sun Yat-sen University (SYSU), and all subjects provided informed consent before the experiments.

### 2.2. Lesions

All patients had subcortical lesions that touched the basal ganglia and the internal capsule and in some patients extended towards occipital and frontal regions. Data from right hemisphere stroke patients were flipped in that all patients displayed their lesion in the left hemisphere. Therefore, the lesion maps were demonstrated precisely and compared directly for left to right hemispheric stroke patients (Figure 1).

### 2.3. Intervention

A virtual environment was displayed on a 42-inch-wide television screen in front of the treadmill. It created the simulations of walking in real-life environment. The scenarios where patients control their gaits consisted of street crossing, park stroll, obstacles striding across, and lane walking. All of the participants received 15 sessions of VRET training (five sessions per week over a 3-week period). Each session lasted up to 60 minutes with breaks as required. Treadmill velocity started at 0.22–0.40 m/s and was increased when normal step length was observed.

### 2.4. Clinical Outcome Measures

Timed 10-meter walk test and fMRI data were collected within 3 days before the commencement of training (pre) and right after the last training session (post).

Gait speed was measured by 10 meters (m) timed walk. Participants were asked to walk at a comfortable speed with or without an assistive device. The average speed of two tests was included in data analysis.

Lower limb impairment and balance were measured by the Fugl-Meyer Assessment: Lower Extremity (FMA-LE) [24] and the Brunel balance assessment scale [25, 26]. Measurements were recorded in stroke subjects at baseline and after 3 weeks of training by an experienced examiner.

### 2.5. fMRI Data Acquisition

fMRI was performed on a 3.0 T scanner (Siemens, Trio Tim, Germany) equipped for echo planar imaging. A 3D, high-resolution, T1-weighted data set of the entire brain was acquired for each subject (TR = 1460 ms, TE = 2.54 ms, field of view 214 × 245, matrix 256 × 256, and a slice thickness of 1 mm). Care was taken to cover all critical brain regions. For fMRI studies, blood oxygen level-dependent weighted scans (TR = 2000 ms, TE = 25 ms, field of view 200 × 200, matrix 64 × 64, and a slice thickness of 3 mm) were acquired.

The fMRI data collection protocol involved five active movement blocks for each participant. Each block was triggered by an auditory command. Active movement blocks were alternated with interspersed periods of absolute rest (20 seconds each). The total scanning time for unilateral movement of one foot was approximately 200 seconds. The activation task was repetitive active ankle dorsiflexion of the unilateral ankle in a purpose-built ankle-foot orthosis. The orthosis permits 5° of plantar flexion and 10° of dorsiflexion. A metronome was used as audio command to pace the movements (30 beats/min = 0.5 Hz).

Prior to scanning, each participant was asked to practice the movement requirements to ensure consistency. Participants' heads were stabilized with straps on a foam-cushioned holder to minimize head motion. The knees were flexed to approximately 135° by placing a soft roll beneath the knees. Both arms were stabilized to minimize movements. Verbal instructions were given to participants to close their eyes during the scan and not to think about ankle movements when at rest.

### 2.6. Data Analysis and Statistics

Imaging data was analyzed using Statistical Parametric Mapping (SPM8; http://www.fil.ion.ucl.ac.uk/spm/software/spm8) implemented in MATLAB 7.0 (Mathworks, Natick, MA, USA). First, all volumes were realigned spatially to the middle volume after slice timing to correct for residual head movement. Any participant with head translations greater than 3 mm for any task condition was excluded from the study. Afterwards, all functional scans were normalized into the standard anatomic space template defined by the atlas of Talairach. Images were spatially smoothed using a Gaussian kernel of 8 mm full-width half-maximum. Functional and structural images of participants with right hemispheric strokes were flipped from right to left so that the image of the left hemisphere represented the lesioned hemisphere. The affected ankle was therefore always the “right” one.

Using image analysis and general linear model statistics (SMP8, random effect module), single-subject contrasts were analyzed in the first-level analysis and then used in a second-level analysis for random effect analyses (one-sample *t*-test and paired *t*-test) to create group maps (*p* < 0.01, uncorrected for multiple comparisons across the whole-brain volume, and extent threshold = 20 voxels) separately for the different groups at each time point. Data analysis was performed by modeling the active and resting conditions as reference waveforms (box-car functions). Five regions of interest were selected: SMC (corresponding to paracentral lobule), SMA, the cingulate motor area, the anterior and posterior cerebellum, and the secondary somatosensory area.

Data analysis was performed in SPSS (version 17.0). Descriptive statistics were used to describe the demographics and gait parameters. Paired *t*-test was used to evaluate the differences of walking ability and clinical scales between pre- and postintervention. Pearson correlation coefficients were computed to test for a relationship between the changes in 10 m walk time and brain activations in the regions of interest. Statistical significance was set at 0.05.

## 3. Results

### 3.1. Effect of VRET on Gait Parameters

The mean 10 m timed walk reduced from 27.78 ± 10.45 seconds to 17.84 ± 5.26 (*p* < 0.05) postintervention. Walking speed increased from 0.40 ± 0.12 m/s to 0.60 ± 0.15 m/s (*p* < 0.05) postintervention. Fugl-Meyer scales showed a significant increase from 23.38 ± 4.03 to 25.38 ± 4.1 (*p* = 0.035) after the training. But there is no significant difference of balance function from Brunel scales (Table 2).

### 3.2. Cerebral Reorganization

**Figure 2:**
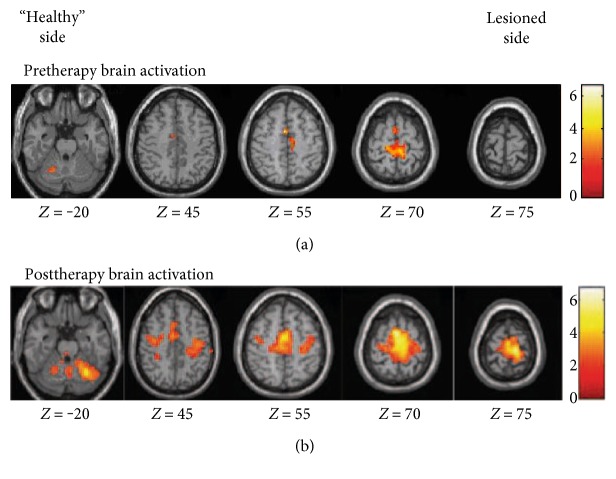
Areas activated by active dorsiflexion: (a) active tasks of the paretic foot pre, (b) post-VR + BWSTT. The lesioned side is on the left of the image.

**Figure 3:**
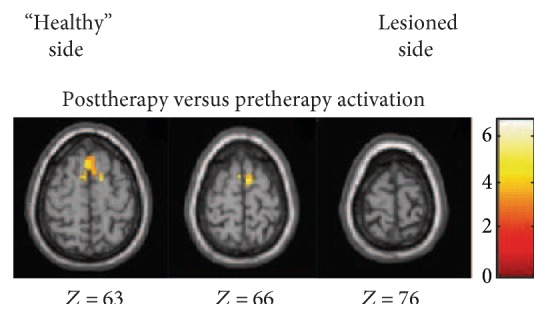
Areas activated by active dorsiflexion: post- versus pre-VR + BWSTT. The lesioned side is on the left of the image.

During the active task performed at baseline with the affected foot, the SMC, the SMA and supramarginal gyrus contralateral and ipsilateral to the movement, and posterior cerebellum ipsilateral to foot movement were activated (Figure 2(a) and Table 3). During the active task performed at postinterventions with the affected foot, the SMC, the SMA, the cerebellum, cingulate motor area, and the supramarginal gyrus contralateral and ipsilateral to the movement were activated (Figure 2(b) and Table 3). At the second measurement, increased neural response of the SMC on the ipsilesional hemisphere and bilateral SMA revealed reorganization of the sensorimotor network (Figure 3 and Table 3). No region was observed to decrease after VR-based training. No mirror movement was observed during fMRI scanning by visual inspection.

**Figure 4:**
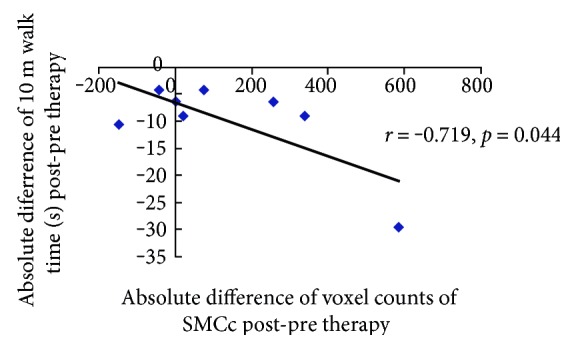
Region of interest analyses. Scatterplot with a linear-fitted regression demonstrating a significant correlation between the voxel count changes from pre to post with movement of the paretic foot versus rest in the lesioned SMC and the absolute decrease in 10 m walk time.

There were no areas with significant changes of activation from pre to post associated with active movement of the unaffected foot versus rest. Brain activity with active movement of the paretic foot versus rest showed a negative correlation (*r* = −0.719, *p* = 0.044) between voxel change in SMC of the lesioned hemisphere and the decrease in time to complete the 10-meter walk after intervention (Figure 4).

## 4. Discussion

This study investigated the therapy-induced plasticity in patients who suffered subacute ischemic stroke by using fMRI. After VRET for three weeks, our recruited subjects demonstrated improvement of walking speed and lower extremity motor function. In the current fMRI study, as a first step to explore the neural correlates of VRET, we investigated that the increased activation in cortical regions of stroke survivors is associated with better walking function.

## 5. Gait Parameters

Gait speed is a reliable measurement of walking ability [23]. This study observed an increase in gait speed of greater than 0.16 m/s which exceeded the minimal clinically important difference previously reported [27]. At an early stage after stroke, similar gains were seen on the Fugl-Meyer and Berg balance scales in both groups [28, 29]. The results of this current study are consistent with published studies that early intervention can improve on balance and lower extremity motors functions in patients with subacute stroke. Findings of this study are consistent with previous studies that VR-enhanced treadmill training which also revealed improvement of gait function for individuals with stroke [7–9, 14]. When VR was combined with treadmill training, the speed of the patient's viewpoint motion in the virtual environment is matched to the speed of the treadmill. Patients receive visual feedback which is close to real-life experience [7]. This combination provided patients after stroke with defined goals and a sense of accomplishment, and their neuroplasticity increased through repetitive exercises of lower extremities, resulting in improved gait ability [11, 12].

## 6. Cortical Reorganization

VR training provided patients with different motor sensory stimulations, which is needed in neural reorganization in the brain [30]. Multisensory (visual and auditory) feedback provided by VR systems allows the central nervous system to better control the position and orientation of body segments [5]. The degree to which motor ability is regained depends on the size of neuronal populations reorganize induced by interventions. Before training, the bilateral SMC and SMA were activated (Figure 2(a) and Table 3). Subactue stroke patients may have increased activation in SMC and SMA after receiving three weeks of treadmill-enhanced VR training (Figure 3 and Table 3). The fMRI data recorded in this study is consistent with other report of cortical contribution in poststroke functional recovery [3]. You et al. [22] found that, however, before the VR training, the ipsilateral SMA, along with the bilateral primary motor cortex and SMC, was activated but was suppressed after VR training. Differences between our design and those of You et al. [22] (such as subacute ischemic stroke patients versus chronic ischemic or hemorrhage stroke patients and treadmill-enhanced VR training versus the IREX VR system) may have led to the distinctions in the observed results. Moreover, the addition of VR may have contributed to the significant improvements seen in the participants by keeping them concentrating more intently on the task so that increased motor learning occurred [6, 8]. Patients in this study increased the gait velocity and decreased the 10 m walk time, but we need further study to investigate the exact underline mechanism.

In this study, the phase of reorganization showed hyperactivation in ipsilesional SMC (Figure 3 and Table 3). Improved SMC activation in the affected hemisphere is one of the common mechanisms underlying functional recovery of the paretic limbs [31]. Expansion of SMC activation after stroke probably reflect the “unmasking” of preexisting but normally inactive representations or “recruitment” of neurons/connections not normally devoted to this function [32, 33]. Repetitive practice of the affected limb may increase efficacy of existing synapses and facilitate synaptic proliferation and axonal sprouting from surviving neurons, thus increase neuroplasticity and associated motor improvement [34].

Another finding was the recruitment of SMA after VRET training (Figure 3 and Table 3). The SMA plays a crucial role in the synchronization of bimanual movements [35]. Recent study suggested that primary motor cortex had a crucial role along with SMA during the motor execution task [36]. Enhancement of SMA activity could benefit primary motor cortex dysfunction in stroke survivors [37]. fMRI study of healthy subjects showed that VR induces activation in brain areas associated with motor control, including the SMA, the inferior parietal cortex, and the inferior frontal cortex [10]. This study suggested that an increase in cortical activation after therapy potentially reflecting the fact that cortical networks may be involved in mediating the effects of treadmill-enhanced VR training.

Neuroimaging findings suggested that VR could induce cortical reorganization of the neural locomotor pathways [10, 22, 23, 25]. This cortical reorganization was associated with notable gain in locomotor function [22, 25]. For example, You and coworkers investigated the correlation between remodeling of the brain and recovery of lower limb function of patients with stroke after VR training, and they found that VR could induce cortical reorganization from aberrant ipsilateral to contralateral SMC activation. Similar to our findings which further included a measure of voxel counts of SMC and SMA and correlated gait functions, we think this kind of enhanced cortical reorganization might play an important role in the recovery of functional ambulation in patients with stroke. In addition, the addition of VR may have contributed to the significant improvements seen in the participants by keeping them concentrating more intently on the task and providing extrinsic motivation.

VRET training may activate many brain regions associated with motor skills and related experiences. According to our results, there exist correlations between the increase of voxel counts in the SMC of the affected hemisphere and the improvement of the time to walk 10 m. The greater the activation increased in lesioned SMC, the better the ambulation recovery (Figure 4). Activation in the contralesional SMC did not correlate with positive recovery. The correlation results suggested that cortical networks could be the site of plasticity or compensatory activation, and their recruitment may be one mechanism by which treadmill-enhanced VR training improves walking in hemiparetic stroke.

## 7. Limitation

This study has several limitations which need cautions for the interpretation and generalizability of the data. One of the limitations was that the sample size was relatively small (*n* = 8) and the recruited subjects in this study have high gait functioning level and could perform voluntary ankle movements in fMRI. Further researches are needed both to enlarge the sample size and to study whether patients with severe functional impairments could also benefit from VRET. Second, the key focus of this preliminary study was to assess if VRET treatment program can induce brain activity changes in subacute stroke survivors and then there was a lack of a control group and limited for confident conclusions. However, previous studies [31] suggested that brain activity changes associated with “spontaneous” recovery and those with training for rehabilitation may be different. The former after stroke is associated with decreased activation in brain motor control regions, whereas increase activation in specific regions within the broader control network accompanies performance gains after rehabilitation poststroke. Our results were consistent with this notion and observed increases in activation with improved gains after VRET training. Meanwhile, previous animal studies have demonstrated that induction of proteins is associated with endogenous neural repair within the first 2 weeks after an ischemic insult [38]. Our study focused on the subacute stroke patients from 3 weeks poststroke to 12 weeks poststroke. The functional recovery in our patients may be more involved in the effect of therapeutic intervention. Moreover, we drew our conclusion based on comparing our results with previous studies. Further randomized control trial with large sample size might warrant if research studies target on comparison of treatment effects of the VRET to conventional PT and/or natural recovery associated changes in brain activity.

## 8. Conclusions

Clinically meaningful improvements in measures of walking ability were found in post-subacute stroke subjects after 3 weeks of virtual reality-enhanced treadmill training. VRET training effect might be associated with increased activation of cortical networks participating in the voluntary ankle dorsiflexion, as displayed by fMRI. SMC recruitment was correlated with decreases in the patient's 10 m walk time. Taken together, these findings gave some clues for the clinical rehabilitation management and the potential mechanisms that VRET may be a suitable therapy for subacute ischemic stroke patients with abnormal gait patterns and poststroke cerebral plasticity could be augmented through the use of VRET.

## Figures and Tables

**Table 1:** Clinical and demographic characteristics.

Patient ID	Age (years)	Sex	Site of lesion	Time from stroke to first fMRI data (days)
1	67	F	L corona radiate-basal nucleus	18
2	51	M	R corona radiate and parietal-occipital-temporal lobe	39
3	67	F	L corona radiate-centrum semiovale and frontal-parietal lobe	69
4	61	M	L corona radiate-basal nucleus	47
5	72	M	R corona radiate-basal nucleus	48
6	59	M	L corona radiate-centrum semiovale	44
7	41	M	R thalamic and posterior limb of the internal capsule	35
8	49	M	R basal nucleus and frontal-insular-occipital lobe	57
Mean ± SD	58.38 ± 9.91			42.25 ± 14.86

F: female; M: male; R: right; L: left; MMSE: Mini-Mental State Examination.

**Table 2:** Walking parameters and clinical scale changes for stroke survivors.

	Before VRET	After VRET	*p* value
10 m walk time (s)	27.78 ± 10.45^∗^	17.84 ± 5.26^∗^	*p* < 0.05
Gait velocity (m/s)	0.40 ± 0.12^∗^	0.60 ± 0.15^∗^	*p* < 0.0001
Fugl-Meyer	23.38 ± 4.03^∗^	25.37 ± 4.1^∗^	*p* = 0.035
Brunel	13.25 ± 0.89	13.63 ± 0.52	*p* = 0.197

^∗^ *p* < 0.05 between pre- and posttherapy for the patient group.

**Table 3:** Significant activated areas (*p* < 0.01, corrected for multiple comparisons across the whole brain volume) during active movement of the paretic ankle and areas with a significant difference in activation (*p* < 0.01) between pre- and posttherapy.

Activated areas	Maximum *Z*-score	MNI coordinates		
		*x*	*y*	*z*
*Pretherapy*				
SMC				
Ipsilesional	3.53	−9	−33	72
Contralesional	2.77	3	−36	69
SMA				
Ipsilesional	2.37	−2	0	63
Contralesional	3.02	6	0	54
Posterior cerebellum				
Contralesional	2.95	24	−63	−15
Supramarginal gyrus				
Ipsilesional	3.61	−63	−30	30
Contralesional	2.60	54	−29	26
*Posttherapy*				
SMC				
Ipsilesional	3.64	−9	27	76
Contralesional	3.04	2	−24	72
SMA				
Ipsilesional	3.50	−2	−3	60
Contralesional	3.43	6	0	63
Cingulate motor area				
Ipsilesional	2.39	−3	−3	48
Contralesional	2.31	6	0	42
Posterior cerebellum				
Ipsilesional	3.01	−27	−60	−24
Contralesional	3.09	21	−63	−15
Anterior cerebellum				
Ipsilesional	2.53	−15	−48	−18
Contralesional	2.50	18	−54	−15
*Posttherapy versus pretherapy*				
SMC				
Ipsilesional	2.60	−12	−18	76
SMA				
Ipsilesional	2.66	−6	0	66
Ipsilesional	2.97	6	6	63

## References

[1] Chen G., Patten C., Kothari D. H., Zajac F. E. (2005). Gait differences between individuals with post-stroke hemiparesis and non-disabled controls at matched speeds. *Gait & Posture*.

[2] Goldie P. A., Matyas T. A., Evans O. M. (2001). Gait after stroke: initial deficit and changes in temporal patterns for each gait phase. *Archives of Physical Medicine & Rehabilitation*.

[3] Enzinger C., Dawes H., Johansen-Berg H. (2009). Brain activity changes associated with treadmill training after stroke. *Stroke*.

[4] Visintin M., Dawes H., Johansen-Berg H. (1998). A new approach to retrain gait in stroke patients through body weight support and treadmill stimulation. *Stroke; a Journal of Cerebral Circulation*.

[5] Lord S. E., Rochester L., Weatherall M., McPherson K. M., McNaughton H. K. (2006). The effect of environment and task on gait parameters after stroke: a randomized comparison of measurement conditions. *Archives of Physical Medicine & Rehabilitation*.

[6] Holden M. K. (2005). Virtual environments for motor rehabilitation: review. *Cyberpsychology & Behavior the Impact of the Internet Multimedia & Virtual Reality on Behavior & Society*.

[7] Feasel J., Whitton M. C., Kassler L., Brooks F. P., Lewek M. D. (2011). The integrated virtual environment rehabilitation treadmill system. *IEEE Transactions on Neural Systems & Rehabilitation Engineering a Publication of the IEEE Engineering in Medicine & Biology Society*.

[8] Walker M. L., Ringleb S. I., Walter R., Crouch J. R., Van Lunen B., Morrison S. (2010). Virtual reality-enhanced partial body weight-supported treadmill training poststroke: feasibility and effectiveness in 6 subjects. *Archives of Physical Medicine & Rehabilitation*.

[9] Kim N., Park Y. H., Lee B. H. (2015). Effects of community-based virtual reality treadmill training on balance ability in patients with chronic stroke. *Journal of Physical Therapy Science*.

[10] Prochnow D., Bermúdez i Badia S., Schmidt J. (2013). A functional magnetic resonance imaging study of visuomotor processing in a virtual reality-based paradigm: rehabilitation gaming system. *European Journal of Neuroscience*.

[11] Cho K. H., Kim M. K., Lee H. J., Lee W. H. (2015). Virtual reality training with cognitive load improves walking function in chronic stroke patients. *Tohoku Journal of Experimental Medicine*.

[12] Kim H., Choi W., Lee K., Song C. (2015). Virtual dual-task treadmill training using video recording for gait of chronic stroke survivors: a randomized controlled trial. *Journal of Physical Therapy Science*.

[13] Yom C., Cho H., Lee B. H. (2015). Effects of virtual reality-based ankle exercise on the dynamic balance, muscle tone, and gait of stroke patients. *Journal of Physical Therapy Science*.

[14] Yang S., Hwang W. H., Tsai Y. C., Liu F. K., Hsieh L. F., Chern J. S. (2011). Improving balance skills in patients who had stroke through virtual reality treadmill training. *American Journal of Physical Medicine & Rehabilitation*.

[15] Cho K. H., Lee W. H. (2013). Effect of treadmill training based real-world video recording on balance and gait in chronic stroke patients: a randomized controlled trial. *Gait & Posture*.

[16] Henderson A., Korner-Bitensky N., Levin M. (2007). Virtual reality in stroke rehabilitation: a systematic review of its effectiveness for upper limb motor recovery. *Topics in Stroke Rehabilitation*.

[17] Crosbie J. H., Lennon S., Basford J. R., McDonough S. M. (2007). Virtual reality in stroke rehabilitation: still more virtual than real. *Disability and Rehabilitation*.

[18] Laver K. E., George S., Thomas S., Deutsch J. E., Crotty M. (2012). Virtual reality for stroke rehabilitation. *Cochrane Database of Systematic Reviews*.

[19] Galvin J., McDonald R., Catroppa C., Anderson V. (2011). Does intervention using virtual reality improve upper limb function in children with neurological impairment: a systematic review of the evidence. *Brain Injury*.

[20] Enzinger C., Johansen-Berg H., Dawes H. (2008). Functional MRI correlates of lower limb function in stroke victims with gait impairment. *Stroke*.

[21] Seitz R. J., Donnan G. A. (2010). Role of neuroimaging in promoting long-term recovery from ischemic stroke. *Journal of Magnetic Resonance Imaging*.

[22] You S. H., Jang S. H., Kim Y. H. (2005). Virtual reality-induced cortical reorganization and associated locomotor recovery in chronic stroke: an experimenter-blind randomized study. *Stroke*.

[23] Tunik E., Adamovich S. V. Remapping in the ipsilesional motor cortex after VR-based training: a pilot fMRI study.

[24] Barbeau H., Visintin M. (2003). Optimal outcomes obtained with body-weight support combined with treadmill training in stroke subjects. *Archives of Physical Medicine and Rehabilitation*.

[25] Schuster-Amft C., Henneke A., Hartog-Keisker B. (2015). Intensive virtual reality-based training for upper limb motor function in chronic stroke: a feasibility study using a single case experimental design and fMRI. *Disability and Rehabilitation: Assistive Technology*.

[26] Dobkin B. H., Firestine A., West M., Saremi K., Woods R. (2004). Ankle dorsiflexion as an fMRI paradigm to assay motor control for walking during rehabilitation. *NeuroImage*.

[27] Tilson J. K., Sullivan K. J., Cen S. Y. (2010). Meaningful gait speed improvement during the first 60 days poststroke: minimal clinically important difference. *Physical Therapy*.

[28] Choi J. H., Han E. Y., Kim B. R. (2014). Effectiveness of commercial gaming-based virtual reality movement therapy on functional recovery of upper extremity in subacute stroke patients. *Annals of Rehabilitation Medicine*.

[29] Song Y. B., Chun M. H., Kim W., Lee S. J., Yi J. H., Park D. H. (2014). The effect of virtual reality and tetra-ataxiometric posturography programs on stroke patients with impaired standing balance. *Annals of Rehabilitation Medicine*.

[30] Gaticarojas V., Méndezrebolledo G. (2014). Virtual reality interface devices in the reorganization of neural networks in the brain of patients with neurological diseases. *Neural Regeneration Research*.

[31] Miyai I., Yagura H., Hatakenaka M., Oda I., Konishi I., Kubota K. (2003). Longitudinal optical imaging study for locomotor recovery after stroke. *Stroke*.

[32] Calautti C., Leroy F., Guincestre J. Y., Baron J. C. (2001). Dynamics of motor network overactivation after striatocapsular stroke: a longitudinal PET study using a fixed-performance paradigm. *Stroke*.

[33] Chen R., Cohen L. G., Hallett M. (2002). Nervous system reorganization following injury. *Neuroscience*.

[34] Liepert J., Bauder H., Miltner W. H., Taub E., Weiller C. (2000). Treatment-induced cortical reorganization after stroke in humans. *Stroke*.

[35] Sadato N., Ibañez V., Deiber M. P., Campbell G., Leonardo M., Hallett M. (1996). Frequency-dependent changes of regional cerebral blood flow during finger movements. *Journal of Cerebral Blood Flow & Metabolism Official Journal of the International Society of Cerebral Blood Flow & Metabolism*.

[36] Bajaj S., Butler A. J., Drake D., Dhamala M. (2015). Brain effective connectivity during motor-imagery and execution following stroke and rehabilitation. *Clinical Neuroimaging*.

[37] Lazaridou A., Astrakas L., Mintzopoulos D. (2013). fMRI as a molecular imaging procedure for the functional reorganization of motor systems in chronic stroke. *Molecular Medicine Reports*.

[38] Biernaskie J., Chernenko G., Corbett D. (2004). Efficacy of rehabilitative experience declines with time after focal ischemic brain injury. *Journal of Neuroscience the Official Journal of the Society for Neuroscience*.

